# Thermal Transport Evolution Due to Nanostructural Transformations in Ga-Doped Indium-Tin-Oxide Thin Films

**DOI:** 10.3390/nano11051126

**Published:** 2021-04-27

**Authors:** Alexandr Cocemasov, Vladimir Brinzari, Do-Gyeom Jeong, Ghenadii Korotcenkov, Sergiu Vatavu, Jong S. Lee, Denis L. Nika

**Affiliations:** 1 E. Pokatilov Laboratory of Physics and Engineering of Nanomaterials, Department of Physics and Engineering, Moldova State University, MD-2009 Chisinau, Moldova; kocemasov@live.ru (A.C.); vbrinzari@mail.ru (V.B.); ghkoro@yahoo.com (G.K.); 2 Laboratory for Spectroscopy of Condensed Matter Physics, Department of Physics and Photon Science, Gwangju Institute of Science and Technology, Gwangju 61005, Korea; dogyeomjeong@gist.ac.kr (D.-G.J.); jsl@gist.ac.kr (J.S.L.); 3 Physics of Semiconductors and Devices Laboratory, Department of Physics and Engineering, Moldova State University, MD-2009 Chisinau, Moldova; savatavu@yahoo.com

**Keywords:** thermal transport, indium-tin-oxide, thin film, thermoelectrics

## Abstract

We report on a comprehensive theoretical and experimental investigation of thermal conductivity in indium-tin-oxide (ITO) thin films with various Ga concentrations (0–30 at. %) deposited by spray pyrolysis technique. X-ray diffraction (XRD) and scanning electron microscopy have shown a structural transformation in the range 15–20 at. % Ga from the nanocrystalline to the amorphous phase. Room temperature femtosecond time domain thermoreflectance measurements showed nonlinear decrease of thermal conductivity in the range 2.0–0.5 Wm^−1^ K^−1^ depending on Ga doping level. It was found from a comparison between density functional theory calculations and XRD data that Ga atoms substitute In atoms in the ITO nanocrystals retaining Ia-3 space group symmetry. The calculated phonon dispersion relations revealed that Ga doping leads to the appearance of hybridized metal atom vibrations with avoided-crossing behavior. These hybridized vibrations possess shortened mean free paths and are the main reason behind the thermal conductivity drop in nanocrystalline phase. An evolution from propagative to diffusive phonon thermal transport in ITO:Ga with 15–20 at. % of Ga was established. The suppressed thermal conductivity of ITO:Ga thin films deposited by spray pyrolysis may be crucial for their thermoelectric applications.

## 1. Introduction

Indium oxide-based compounds and composites with high thermal stability have been intensively investigated as prospective semiconducting materials for high-temperature thermoelectric applications. Additives or supplement oxides of different metallic nature like Zn, Sn, Ge, Ga, Y, Nb, Ni etc. were used [1,2,3,4,5,6,7] in such compounds and composites to improve their thermoelectric and optical properties. Some of these materials retained the lattice structure of the host oxide at large amounts of additives. The specifics of In_2_O_3_ lattice are its complexity and large number of atoms in the primitive cell (40). As a consequence one could expect the reduction of phonon thermal conductivity $\kappa_{ph}$ due to the trapping of heat energy by numerous optical modes and flattening of their energy dispersions [8], leading to lower phonon group velocities. The structural complexity of Ia-3 space group impedes investigations of various defect complexes in In_2_O_3_ and their impact on electronic, phononic and thermoelectric properties. In Ref. [9], some of us reported on the theoretical investigations of electronic properties of In_2_O_3_ with Sn-, Ga- and O-based point defect complexes, employing a density functional theory. It has been shown that defect complexes strongly influence the electronic band structure and position of indium and oxygen atoms [9]. Defect complex driven deviation in both atomic mass and crystal lattice strain results in enhancement of phonon scattering by point defects and may affect the lattice thermal conductivity. The impact of different defect complexes and structural vacancies on thermal properties of In_2_O_3_ requires additional investigations.

The strong improvement of specific electronic, thermal and thermoelectric properties could be reached when the material is in nanocrystalline form. Recent theoretical results demonstrated that enhanced electrical conductivity σ and thermopower *S* providing an elevated power factor $PF=\sigma S^{2}$ can be achieved in ITO nanofilms [10] due to the filtering effect [10,11] of low energy conduction electrons at grain boundaries. *PF* values of around 3 mW·m^−1^·K^−2^ were reported for thermally aged ITO films with an optimal Sn content [12,13]. Thorough independent experimental study [14] confirmed the presence of potential barrier in the vicinity of grain boundaries which are responsible for the filtering of electrons despite the degeneracy of ITO conduction band. ITO nanofilms have also demonstrated reduced values of thermal conductivity as compared with bulk ITO [15]. The drop of thermal conductivity was explained by strong phonon scattering on grains, reduction of electronic part $\kappa_{el}$ of thermal conductivity due to the filtering of low energy conduction electrons and porosity of ITO films [15]. Due to the geometric complexity of the nanograin network, the trapping of heat carrying vibrational modes leading to the decrease of phonon thermal conductivity $\kappa_{ph}$ is also possible. Reduction of thermal conductivity without degradation of electronic parameters (σ, *S*) radically improves the thermoelectric efficiency, determined by figure of merit $ZT=\sigma S^{2}T/\kappa_{tot}$. Looking for compounds with low $\kappa_{tot}$ and high enough σ and *S* is a mainstream in thermoelectric material science. The strong suppression of heat conduction in different nanolayers, nanowires and superlattices due to phonon interface scattering and/or spatial confinement of phonons was reported in numerous theoretical and experimental studies [16,17,18,19,20].

Our experiments with ITO:Ga thin films deposited by spray pyrolysis have shown quite large values of *PF* [13]. However, reported values of electrical conductivity were smaller than those in undoped ITO due to the peculiarities of spray pyrolysis technique used, leading to the formation of finely dispersed Ga_2_O_3_ grains on the main crystallite surfaces [13]. At the same time the influence of deposition parameters (like as pyrolysis temperature) on electric and thermal properties of such films requires further investigations and optimization. ITO films remain totally nanocrystalline up to 50 at. % Sn [12], while nanocrystalline to amorphous phase transition in ITO(Sn ≤ 10 at. %):Ga-based films occurs at Ga content ≥ 20–30 at. % and may lead to a sharp drop in the thermal conductivity.

Thereby, in this work we focus on experimental and theoretical investigations of thermal conductivity in ITO:Ga thin films with widely varying Ga content and almost constant amount of Sn ~6 at. %. Such concentration of Sn [12,13] is optimal in terms of *PF* and *ZT*. The experiments were performed by an ultrafast (femtosecond) laser-induced time domain thermoreflectance method (TDTR) [21,22]. It is a powerful and versatile technique for thermal properties investigations of a large variety of bulk and nanoscale systems. Within the many advantages of TDTR in comparison with conventional thermal conductivity measurements is an excellent spatial resolution at a length scale below tens of micrometers. TDTR also requires minimal sample preparation for the measurements. Using density functional theory (DFT) and linearized Boltzmann transport equation (BTE) for phonons, we investigate in detail how changes in phonon energy spectra affect both phonon scattering mechanisms and lattice thermal conductivity in ITO:Ga films at different Ga concentrations.

The rest of the paper is organized as follows. In Section 2 we discuss sample preparation and characterization. Section 3 describes TDTR technique of thermal conductivity measurements. Our theoretical model used for the calculations of phonon modes and lattice thermal conductivity in ITO:Ga films is presented in Section 4. Discussions of results are provided in Section 5. Conclusions are given in Section 6.

## 2. Sample Preparation and Characterization

ITO films (6 at. % of Sn content in sprayed solution) were deposited at T = 350–360 °C by spray pyrolysis method on polished silicon substrates (1 × 1 cm^2^). Additionally, within the same deposition procedure, several films were doped by Ga at various concentrations in the range up to 30 at. %. Hereinafter, under Ga concentration we refer to the following ratio: [Ga]/([In] + [Sn] + [Ga]) where corresponding partial concentrations of compound are indicated in parentheses. The sprayed solution was prepared as a mixture of 0.2M precursors of InCl_3_, SnCl_4_·5H_2_O and GaCl_3_ by dissolving in the dimethylformamide (DMF). The required film thickness around 200 nm was roughly monitored during the deposition of the film by its color and by sprayed solution volume and then was measured by F20 Filmetrics instrument from KLA (San-Diego, CA, USA). All experimental samples were annealed for a stabilization of film structural parameters at T_an_ = 500 °C during half an hour. Other details of our preparation method are provided in Ref. [23].

The deposited films were characterized for their structural properties by an EMPYREAN XRD system from Malvern Panalytical (Almelo, the Netherlands) using Cu Kα (λ = 1.5405 Å) radiation within a diffraction angle range of 15–70° in θ/θ mode. The surface roughness was measured by atomic force microscopy (AFM) using a Park XE7 instrument (Park System corp., Suwon, Korea). Average surface roughness of deposited films did not exceed 10 nm. Surface images of the films were obtained by scanning electron microscopy (SEM) using the Hitachi S-4700 instrument (Hitachi High-Tech corp., Tokyo, Japan). The films were monitored by using conventional secondary electrons mode at an accelerating voltage of 10 kV. 

**Figure 1:**
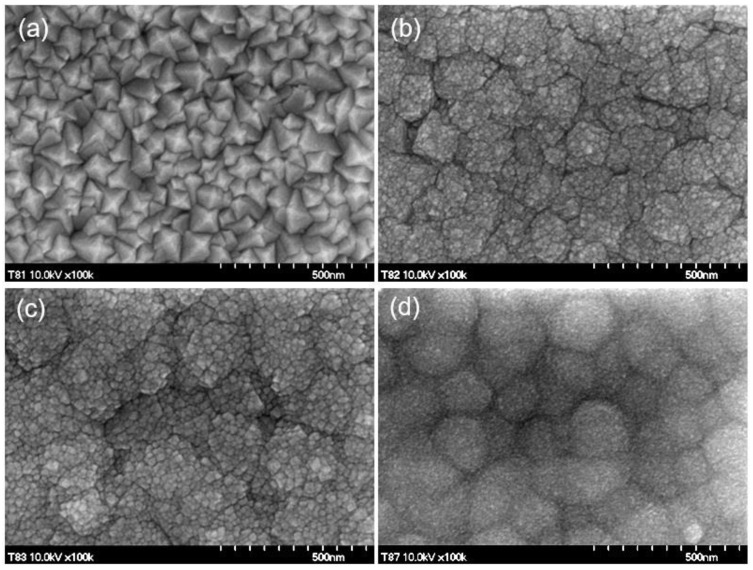
(**a**–**d**). SEM images of ITO:Ga films with 0, 5, 10 and 15 at.% Ga.

Figure 1a–d presents SEM images for ITO:Ga films with Ga content of 0, 5, 10 and 15 at. %. Morphological characteristics of films change significantly with increasing Ga doping. Such evolution is related to peculiarities of grain growth. The thermal yield of the pyrolysis reaction is sensitive to the chemical nature of precursor and strongly influences the local instantaneous crystallization temperature. The latter affects the morphology of the film [23,24,25,26]. We revealed that addition of GaCl_3_ in a spray solution causes a substantial decrease of the droplet size of the aerosol, leading to an increase of the density of crystallization centers and the number of growing grains with corresponding decrease of their size. Besides that, the growth stage with simultaneous adding of Sn and Ga is accompanied by a decrease of the oxide formation energy [9] and its lattice constant (3–7% bond contraction), resulting in a lattice distortion and appearance of various defects. Rise of defect concentration and increase of surface tension of grains also diminish their effective size. The fundamental principle of minimum surface energy for the system of grains results in various growth effects: from crystallites twinning to their agglomeration. It follows from Figure 1 that such processes begin already at 5 at. % Ga.

**Figure 2:**
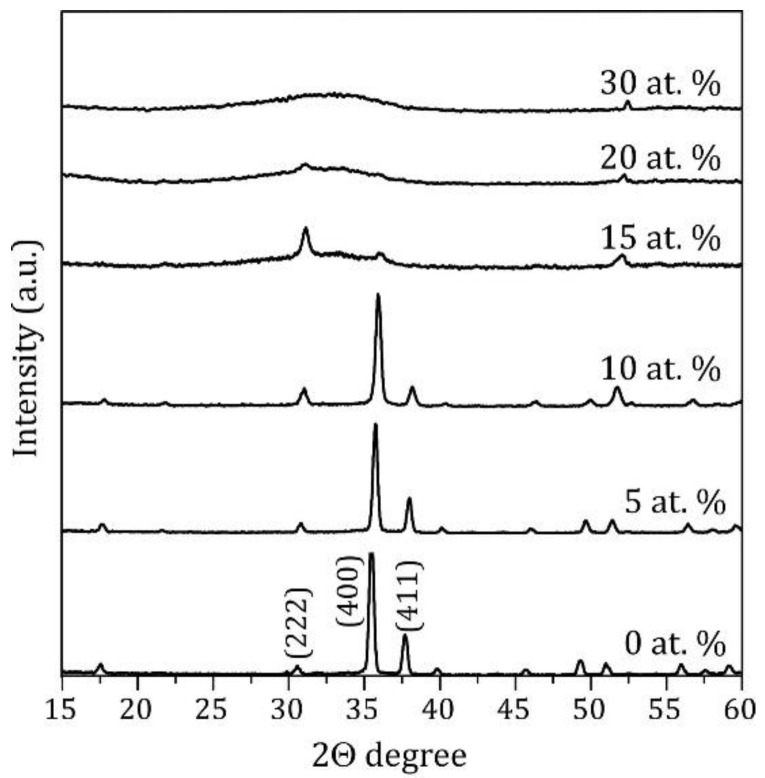
XRD intensity pattern for ITO:Ga films.

At Ga concentrations more than 15 at. %, the films demonstrated a totally amorphous structure. The XRD summary pattern for all samples with tested Ga contents shown in Figure 2 confirms this conclusion. The In_2_O_3_ characteristic peaks practically disappear at 20 at. % Ga. At the range 0–15 at. % of Ga, XRD 2θ diffractograms show the existence of In_2_O_3_ nanocrystalline phase, although it does not imply the absence of finely dispersed Ga_2_O_3_ phase.

These two methods allow to compare polycrystalline effective grain sizes in cross-plane (extracted from XRD pattern by Scherrer formula) and in-plane (extracted from SEM images) directions, respectively.

**Figure 3:**
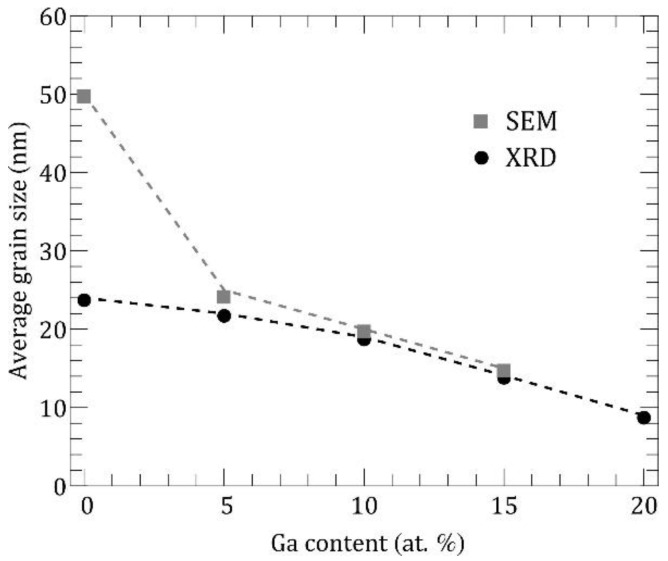
Average grain size in ITO:Ga thin films. Squares and circles denote SEM and XRD data, respectively.

In Figure 3 we show corresponding values of average grain sizes versus Ga content in the measured films. The difference in grain sizes extracted from XRD and SEM patterns for pure ITO film reflects the anisotropy growth during deposition that is peculiar for large enough grains with surface faceting. The Ga additive manifests in smaller round-shaped nanocrystallites which demonstrate a tendency to agglomeration. The position of dominated XRD In_2_O_3_ peaks and their shift with Ga content allowed us to calculate the changes in the lattice constant.

## 3. TDTR Measurements

The thermal transport properties were examined by femtosecond laser-induced time domain thermoreflectance method. An Al film was pre-deposited on the ITO:Ga films, and it was used as the thermal transducer of femtosecond laser pulses. The pump beam was modulated by an electro-optic modulator at 10 MHz and illuminated the transducer with a Gaussian radius of 5.9 μm. The pump-induced reflectivity change was monitored by the probe beam of which amplitude was collected with a fast amplified silicon detector connected to a lock-in amplifier. We obtained in-phase (V_in_) and out-phase (V_out_) profiles of the pump-beam-induced reflectivity variation, of which phase was compared to the internal sinusoidal reference signal of the lock-in amplifier. We fitted the TDTR signal of -V_in_/V_out_ in a temporal range between 100 ps and 3.75 ns by considering the thermal transport model in multi-layered geometry based on the Fourier’s law [21]. We set the boundary conductance across the Al-ITO interface G_Al-ITO_ and thermal conductivity of ITO films κ_ITO_ as two adjustable parameters. Heat capacities of Al (*C*_Al_ = 2.42 J cm^−3^ K^−1^), Si (1.64 J cm^−3^ K^−1^) and ITO (*C*_ITO_ = 2.78 J cm^−3^ K^−1^) were taken from the literature [27,28]. We used bulk value of ITO heat capacity since the spatial phonon confinement in the considered ITO films with thicknesses ~200 nm is weak. Moreover, a preliminary theoretical study, employed an elastic continuum model, suggested that the heat capacity in the very thin silicon membranes is the same as in bulk silicon [29]. The thermal conductivity of the Al transducer was obtained from the direct current electric conductivity by using a Wiedemann-Franz law. The Al film thickness was estimated to have an average thickness of 100 ± 3.6 nm by taking the position of an acoustic echo peak in the TDTR signal. The oscillating thermal wave cannot reach the bottom silicon substrate because of the very low thermal diffusivity of ITO:Ga thin films. The average oscillating heat penetration depth was estimated to be within 34–65% of ITO:Ga thickness. Because the lock-in amplifier independently captures and separates the oscillating thermal responses from steady-state heating, the thin films investigated here can be considered to be semi-infinite.

**Figure 4:**
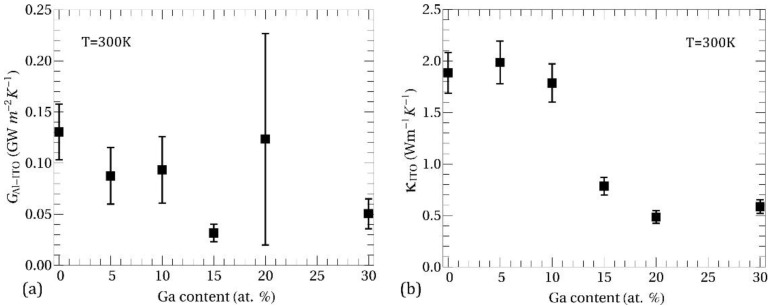
(**a**) Thermal boundary conductance across the Al-ITO interface. (**b**) Thermal conductivity of ITO thin films.

To check the validity and credibility of obtained thermal boundary conductance and thermal conductivity, we examined how the final modeling results are sensitive to those parameters in the given measurement configuration. The sensitivity is determined as the relative change of TDTR signal with respect to the relative change of the parameter of interest. We found that our TDTR results were highly sensitive to κ_ITO_, but not to the other parameter G_Al-ITO_. The latter is attributed to the very low thermal diffusivity of ITO samples so that the thermal gradient of oscillating heat is mostly given in ITO. Despite the low sensitivity to G_Al-ITO_, we could successfully determine the quantity since the covariance between the two fitting parameters is small as about 0.2–0.5, except for the Ga content of 20 at. % having the value close to 0.6. In the error estimation, we considered the sensitivity results, uncertainties in the pre-parameters used in the thermal transport model, and the position-dependent inhomogeneity. After merging all these factors, we estimated the final error by minimizing the Kullback-Leibler divergence [30]. The obtained parameters of G_Al-ITO_ and κ_ITO_ are presented in Figure 4a,b, respectively. At 20 at. % of Ga the error bar of κ_ITO_ is relatively small, while that of G_Al-ITO_ is much larger than error bars for other Ga content. The latter is attributed to the large covariance between κ_ITO_ and G_Al-ITO_ and to the high thermal resistivity of the amorphous phase, which allow us to determine κ_ITO_ more reliably than G_Al-ITO_.

## 4. Theoretical Methods

All electronic calculations were performed within density functional theory formalism as implemented in the SIESTA code [31]. A generalized gradient approximation (GGA) for exchange-correlation functional of Perdew, Burke and Ernzerhof (PBEsol) [32] was used. Scalar-relativistic norm-conserving pseudopotentials for core states [33] generated with PseudoDojo [34] and double-zeta basis set for localized atomic orbitals were employed, with the following valence configurations for atoms: In 4s^2^4p^6^4d^10^5s^2^5p^1^, Sn 4s^2^4p^6^4d^10^5s^2^5p^2^, Ga 3s^2^3p^6^3d^10^4s^2^4p^1^ and O 2s^2^2p^4^. A real-space mesh with energy cutoff of 300 Rydberg and 3 × 3 × 3 reciprocal-space mesh of the Monkhorst-Pack type [35] were found sufficient to perform structure optimizations and calculate the second-order interatomic force constants. The force and stress convergence criteria were set to 0.001 eV/Å and 0.005 GPa, respectively. For relaxed In_2_O_3_ with 40-atom primitive cell (In_16_O_24_) we have obtained a lattice constant of 10.048 Å and volumetric density of 7.27 g/cm^3^, which are close to experimental values of 10.117 Å [36] and 7.18 g/cm^3^ [37].

**Figure 5:**
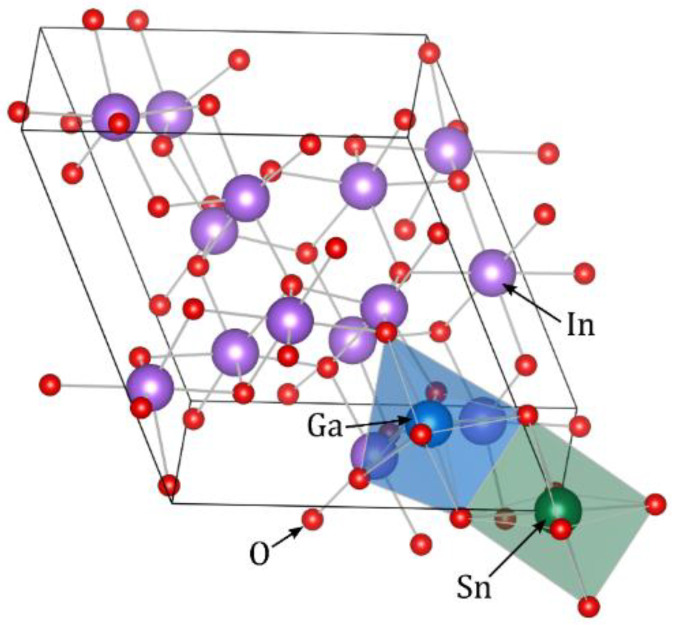
Example of ITO:Ga crystal structure. Two octahedra denote Sn (green) and Ga (blue) substitutional defects surrounded by six nearest oxygen atoms.

The ITO with various Ga doping concentrations was simulated by adding (removing) a certain number of Ga (In) atoms in a 40-atom primitive cell of bixbyite crystal structure, an example of which is shown in Figure 5. In all cells one indium atom at *b*-site was replaced by a tin atom, which corresponds to ITO with 6.25 at. % Sn-doping. The harmonic force constants were calculated using the finite difference method as implemented in the PHONOPY package [38]. The displacement length of each atom from its equilibrium position was set to 0.015 Å.

Once the interatomic force constants were obtained, the dynamical matrix was constructed and diagonalized, thus obtaining phonon dispersions $\omega_{s}\left(q\right)$, where *s* is the phonon branch number and ***q*** is the phonon wavevector. Based on the phonon dispersion data the phonon thermal conductivity was calculated using the linearized Boltzmann transport equation:

(1) $$\kappa_{ph}=\frac{1}{NV}\sum_{q,s}\hbar \omega_{s}\left(q\right)\upsilon_{s,x}^{2}\left(q\right)\tau_{s}\left(q\right)\frac{\partial f}{\partial T}.$$

In Equation (1), summation is performed over entire Brillouin zone mesh and all phonon branches, *T* is the temperature, *N* is the number of *q*-mesh points, *V* is the unit cell volume, ω is the phonon frequency, $\upsilon_{x}=d\omega /dq_{x}$ is the phonon group velocity along the thermal gradient, τ is the phonon lifetime and *f* is the Bose-Einstein distribution function. Converged results for the thermal conductivity were achieved for a dense 20 × 20 × 20 *q*-point mesh (*N* = 8000 points). It is implied in Equation (1) that thermal flux is directed along the temperature gradient, that is, along *X* Cartesian axis. The choice of the latter is formal, since thermal conductivity tensor in bixbyite crystals is isotropic.

In the case of crystalline (nanocrystalline) structures the phonon scattering rate was calculated according to the Matthiessen’s rule:

(2) $$1/\tau =1/\tau_{U}+1/\tau_{GB},$$

where $1/\tau_{U}=BT\omega_{s}^{2}\left(q\right)e^{-1/T}$ is the three-phonon Umklapp scattering rate [39] and $1/\tau_{GB}=\upsilon_{s}\left(q\right)/l_{G}$ is the phonon scattering rate on grain (i.e., nanocrystal) boundaries with the average grain size $l_{G}$. The scattering on grains is assumed to be diffusive, so all incident phonons completely lose their momenta after they reach the grain boundary. Parameter *B* = 0.001 fs/K of the Umklapp scattering was found from the comparison between calculated and experimental [7] thermal conductivities of pure In_2_O_3_, i.e., without phonon scattering on grains.

In the case of amorphous structures, the dominant mechanism of heat transport is diffusion between localized atomic vibrations [40], in contrast to the wave-like phonon transport in crystals. In order to model the diffusive nature of thermal transport in amorphous ITO:Ga we employ here an approach developed by some of us in Ref. [41]. Comparing equations for lattice thermal conductivity within linearized BTE (see Equation (1)) and within a random-walk diffusion theory of heat conduction [42], one can obtain the following expression for the rate of diffusion of lattice vibrations in an amorphous material:

(3) $$1/\tau =\frac{3\pi \upsilon_{s}^{2}\left(q\right)\omega_{s}\left(q\right)}{V_{0}^{2/3}{\langle \omega \rangle }^{2}},$$

where $V_{0}$ is the volume per atom and 〈ω〉 is the mean vibrational frequency, which we have estimated from the condition that 〈ω〉 is the frequency at which integrated phonon density of states (DOS) equals one-half: $\int_{0}^{\langle \omega \rangle }g\left(\omega \right)d\omega =1/2$. In this way we avoided using any free parameters in our thermal conductivity model for ITO:Ga films.

The electronic part $\kappa_{el}$ was estimated according to the filtering model [10,11,43]. Necessary parameters of ITO used in the calculations were taken from [44]. Electron scattering on polar optical phonons and ionized impurities were taken into consideration. The total thermal conductivity was obtained as a sum of the phononic and electronic parts: $\kappa_{tot}=\kappa_{ph}+\kappa_{el}$. Also, the porosity is an inevitable structural factor for spray-pyrolyzed structures. The effect of porosity on the thermal conductivity of ITO:Ga was accounted according to the effective medium theory [45,46]. The porosity of our films was estimated in the range 25–35% by comparing the real refractive index of the films measured by laser ellipsometry with the same one for the bulk material [47]. Such porosity is within a reasonable range, since 44% porosity of In_2_O_3_ [48] was reported for samples synthesized at 100 °C. We note here that we do not clarify an influence of pore ordering and their geometry on $\kappa_{tot}$, since currently we have no technological control over these factors. This question requires an additional investigation and remains to be addressed in the future. Thereby, for all calculated structures the porosity was constant and fixed to 30% to facilitate the theoretical analysis and rather to focus on the understanding how changes in phonon dispersion and scattering mechanisms affect the thermal transport at various Ga concentrations. It is worth noting that higher porosity means lower thermal conductivity, thus providing another way for phonon engineering [49,50,51,52] and tuning the thermoelectric performance of indium oxide-based material.

## 5. Results and Discussion

**Figure 6:**
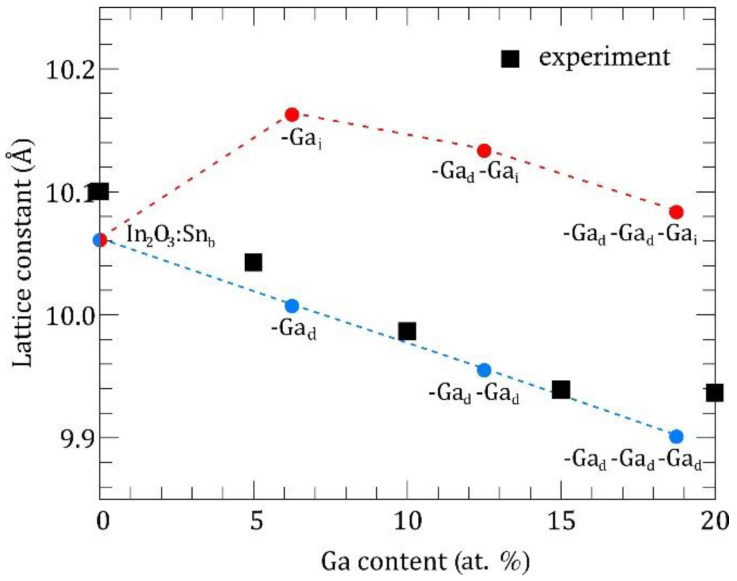
Lattice constant of ITO:Ga as a function of Ga content. Squares denote XRD data. Red and blue circles denote theoretical calculations with Ga atoms placed in different positions (interstitial *c*-site and substitutional *d*-site).

For accurate modeling of the thermal conductivity in our structures we should determine which position of the ITO lattice tend to occupy Ga atoms. For this purpose, we have extracted the lattice constants at different Ga concentrations from our XRD measurements and compared them with theoretical values calculated from DFT relaxation procedure. Figure 6 shows the evolution of the ITO lattice constant as a function of Ga content.

According to our previous work on In_2_O_3_ point defect formation energies [9], there are in general two possible scenarios for Ga atoms. In the first scenario, all Ga atoms occupy substitutional positions (Figure 6, blue circles), which are the most energetically favorable. Here the theory predicts the compression of ITO crystal structure with a linear decrease of lattice constant given by the law: a(x)=a(0)−0.0085x, where a(0)=10.06 Å. In the second scenario, which is less likely from energetical point of view, Ga atom is placed at an intersite (*c*-site, i.e., structural vacancy, specific for Ia-3 space group). In this case calculations predict an expansion of the crystal structure with an increase of the lattice constant from 10.06 Å at 0 at. % Ga to 10.16 Å at 6.25 at. % Ga. However, introduction of additional Ga atoms in substitutional positions results again in the lattice compression (Figure 6, red circles). According to experimental data (Figure 6, squares) the lattice constant decreases when Ga content increases from 0 at. % to 15 at. %, supporting the first theoretical scenario with all Ga atoms forming substitutional point defects. Our study of ITO:Ga system demonstrates that within the given technological conditions the bixbyite lattice structure is maintained until a quarter of In atoms is substituted by guest atoms and no secondary oxide phases (SnO_2_, Ga_2_O_3_) appear with non-bixbyite crystallographic symmetries, which confirms the high stability of cubic In_2_O_3_ structure. On the other hand, such substitution value (~0.25) also means a high solubility limit of Ga atoms in ITO system. Further increase of Ga doping dramatically changes the crystal structure: XRD and SEM measurements (see Figure 1 and Figure 2) show that the structures become amorphous and lose the long-range atomic order. Nevertheless, it means that formation of In-Sn-Ga-O compound occurs even with increased Ga content in the deposited precursor. Otherwise, traces of the bixbyite structure should have been preserved on the XRD spectra.

**Figure 7:**
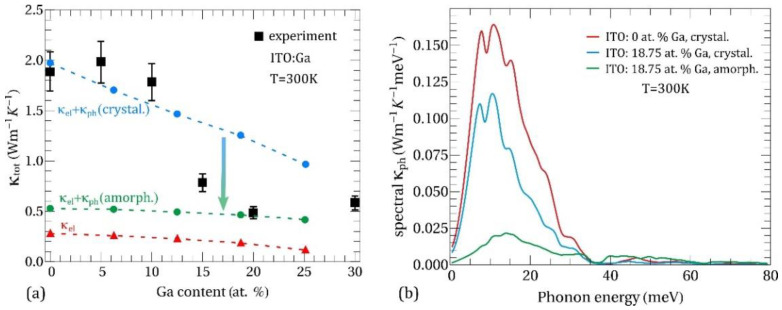
(**a**) Total thermal conductivity of ITO:Ga films at 300 K as a function of Ga content. Squares represent experimental data, while circles denote theoretical calculations with phonon lifetimes for crystalline (blue circles) and amorphous (green circles) materials. Electronic thermal conductivity is denoted by red triangles. (**b**) Spectral phonon thermal conductivity in ITO with 0 at. % Ga (red line) and 18.75 at. % Ga (blue and green lines) as a function of phonon energy. Calculations with “crystalline” and “amorphous” phonon lifetimes are presented.

Figure 7a shows the room temperature total thermal conductivity in ITO:Ga films as a function of Ga content. Squares denote experimental data, while circles represent theoretical calculations of $\kappa_{tot}$ with Ga atoms in substitutional positions. Calculation of $\kappa_{el}$ within filtering model [10,11,43] is also presented for comparison (triangles). Intending to elucidate how the changes in crystalline structure affect the thermal conductivity at different Ga concentrations, we have performed two calculations of $\kappa_{ph}$: (i) with phonon lifetime for crystalline films according to Equation (2) (blue circles in Figure 7a) and (ii) with phonon lifetime for amorphous films according to Equation (3) (green circles in Figure 7a). In general, the experimental results revealed a decreasing trend in $\kappa_{tot}$ for increasing Ga doping up to 15–20 at. %, which reasonably agrees with the calculations employing phonon lifetimes for crystalline films. As the level of disorder increases further (20 at. % and 30 at. % Ga) the thermal conductivity saturates around value ~0.5–0.6 Wm^−1^ K^−1^, which is close to the theoretical predictions with phonon lifetimes for the amorphous case. Thus, our theoretical results suggest that in the range 15–20 at. % Ga there is an evolution from propagative (i.e., crystalline) to diffusive (i.e., amorphous) phonon thermal transport in ITO:Ga films, which is in a good agreement with XRD and SEM measurements showing crystal-to-amorphous structural transition.

**Figure 8:**
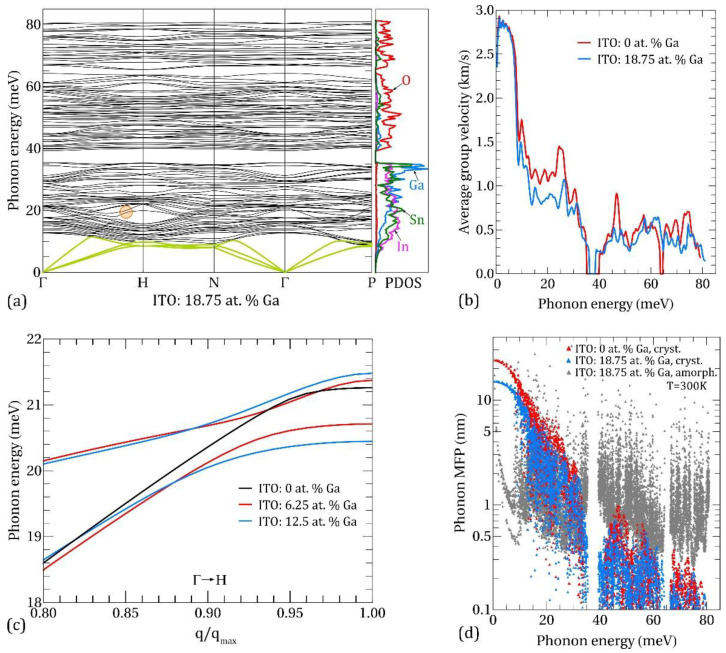
(**a**) Phonon dispersions and PDOS in ITO with 18.75 at. % Ga. The green curves in phonon spectra highlight acoustic branches. The orange circle designates the region with a clear avoided crossing. (**b**) Average phonon group velocity as a function of phonon energy in ITO with 0 at. % Ga (red line) and 18.75 at. % Ga (blue line). (**c**) Avoided-crossing behavior of phonon dispersions in ITO:Ga. (**d**) Phonon mean free paths at 300 K.

At the same time our estimation of the electronic thermal conductivity demonstrates reduction of $\kappa_{el}$ from 0.28 Wm^−1^ K^−1^ at 0 at. % Ga down to 0.19 Wm^−1^ K^−1^ at 18.75 at. % Ga. Amplification of electron scattering arises from the reduction of the grain size with the rise of Ga content. The electron concentration in the conduction band remains unchanged because of the isovalent nature of Ga impurity. The obtained values of $\kappa_{el}$ are in the same range as $\kappa_{ph}$ in amorphous calculation, while they are several times lower than $\kappa_{ph}$ in crystalline calculation. It is clear from Figure 7a that decreasing of the total thermal conductivity of ITO:Ga films as Ga concentration increases up to 15–20 at. % is mainly due to the suppression of the phononic contribution. Specifically, there are two responsible factors: (i) reduction of the average grain size $l_{G}$ (see Figure 3) with the corresponding increase of the phonon scattering rate and (ii) modification of the phonon energy spectra accompanied by the diminution of the average group velocity of phonons (see Figure 8b below). These are the main factors behind the thermal conductivity drop in the nanocrystalline ITO:Ga films.

A useful quantity that reveals the contribution of phonons at various energies into heat conduction is the spectral phonon thermal conductivity $\kappa_{ph}\left(E\right)$, determined by: $\kappa_{ph}=\int \kappa_{ph}\left(E\right)dE$. Figure 7b shows $\kappa_{ph}\left(E\right)$ in crystalline and amorphous calculations for ITO with 0 at. % Ga (red line) and 18.75 at. % Ga (blue and green lines). The largest contribution to $\kappa_{ph}$ comes from phonons with energies in the range 0–35 meV. The contribution of higher energy modes > 40 meV in the amorphous case (green line) is ~25% of $\kappa_{ph}$, while it is almost negligible in the crystalline case (red and blue lines) due to an enhanced phonon-phonon Umklapp scattering characterized by a quadratic dependence on phonon frequency: $1/\tau_{U}\sim \omega^{2}$. Moreover, it is obviously seen that suppression of thermal conductivity with increasing Ga content in the crystalline case is due to the weaker contribution from low-energy (0–35 meV) vibrations. In the case of amorphous films (>15 at. % Ga) the $\kappa_{ph}\left(E\right)$ dependence is almost insensitive to the amount of Ga since according to Equations (1) and (3) $\kappa_{ph}\left(E\right)\sim \langle \omega \rangle$, while mean vibrational frequency 〈ω〉 deviates very weakly for different Ga concentrations e.g., ℏ〈ω〉=46 meV at 0 at. % Ga and ℏ〈ω〉=45 meV at 18.75 at. % Ga.

We now turn to a more detailed analysis of the phonon properties and associated effects behind the thermal conductivity suppression in our ITO:Ga films. The phonon dispersion and projected density of states (PDOS) in ITO with 18.75 at. % Ga is shown in Figure 8a. The PDOS curves are presented per one atom of the respective specimen. The maximum energy of optic phonons is ~80 meV and it is practically independent of Ga doping. Three acoustic branches (light green curves)—one longitudinal acoustic (LA) and two transversal acoustic (TA)—demonstrate a linear dispersion near the Brillouin zone center, with sound velocities: $\upsilon_{LA}$ = 4.7 km/s and $\upsilon_{TA}$ = 2.9 km/s. At 0 at. % Ga, $\upsilon_{LA}$ = 4.8 km/s and $\upsilon_{TA}$ = 3.0 km/s, so one can conclude that rise of Ga content results in a weak softening of the acoustic branches.

According to the dispersion and PDOS data it is natural to split the whole energy range into the lower part (0–35 meV), which is dominated by the vibrations of the metal atoms and the upper part (>40 meV) in which the vibrations of lighter oxygen atoms determine the vibrational spectrum. These two parts are separated by an energy gap in the range ~35–40 meV, which slightly narrows as Ga concentration increases. The low-energy part of the spectra is characterized by hybridization (mixing) of metal-type vibrational modes—mixed In/Sn, In/Ga and Sn/Ga modes. These hybridized modes are accompanied by the appearance of multiple avoided-crossing points throughout the Brillouin zone (see Figure 8c for an example), resulting in the flattening of the dispersion law and a corresponding decrease of the average group velocity at phonon energies between 10 and 30 meV (see Figure 8b). This effect is more pronounced for higher Ga concentrations and it plays an important role in the suppression of the phonon heat conduction in nanocrystalline ITO:Ga films. The similar avoided-crossing behavior was found in “host-guest” type materials such as Ba_8_Ga_16_Ge_30_ clathrate [53] and YbFe_4_Sb_12_ skutterudite [54]. An analytical model describing the phonon dispersion relation of host-guest lattices with heavy guest atoms was recently proposed in Ref. [55].

It is insightful to separate between the intrinsic and extrinsic factors influencing the $\kappa_{ph}$ of ITO:Ga at different Ga-doping. The intrinsic element is the modification of phonon dispersion and group velocities, related to the changes in the atomic composition and interatomic forces. From the extrinsic factors, we can distinguish the variation in average grain size, which directly affects the phonon scattering rate on the grain boundaries. It should be noted that extrinsic factors usually could be tailored within a certain limit by the technological conditions, thus providing additional way for managing the thermal flux in ITO-based structures. In order to quantify the influence of the above factors on $\kappa_{ph}$ we show in Figure 8d the mean free paths (MFPs) of phonons calculated, taking into account Umklapp processes and phonon scattering on the grain boundaries (red and blue triangles). It can be seen that MFPs of low-energy phonons (<10 meV) in nanocrystalline ITO:Ga are mostly limited by the scattering on grains with an average grain size $l_{G}$ = 24 nm for 0 at. % Ga and $l_{G}$ = 15 nm for 18.75 at. % Ga, as was determined from our XRD measurements presented in Figure 3. For higher energies, the phonon-phonon Umklapp scattering prevails and the phonon MFPs quickly drop. Nevertheless, according to the Bose-Einstein distribution the population factors of the low-energy modes are smaller than of higher energy modes, and as a result the overall contribution to the heat flux from the energy range 0–10 meV does not exceed 30% as determined from the spectral $\kappa_{ph}$ data (see Figure 7b). At the same time, phonons with energies 10–35 meV are the main heat carriers at room temperature, transferring more than 2/3 of the total thermal flux. Therefore, the drop of thermal conductivity in nanocrystalline ITO:Ga films as the Ga content increases is primarily due to the redistribution of the vibrational spectrum in this energy range, leading to the diminution of the average group velocity and MFPs of phonons. Finally, in the upper part of the spectra (>40 meV) all modes possess MFPs below 1 nm owing to the low group velocities and enhanced Umklapp scattering, resulting in an almost negligible contribution to the thermal conductivity as follows from $\kappa_{ph}\left(E\right)$ on Figure 7b. Oppositely, the phonon MFPs in an amorphous calculation (gray triangles in Figure 8d) are almost uniformly distributed over entire energy range and this picture is independent of Ga content. It explains the relatively high contribution of high-energy phonon modes in the amorphous case as well as the much weaker dependence of phonon thermal conductivity on Ga amount in comparison with nanocrystalline ITO:Ga films.

## 6. Conclusions

Thermal properties of indium-tin-oxide thin films with Ga concentrations up to 30 at. % deposited by spray pyrolysis were investigated both experimentally and theoretically. XRD and SEM showed the existence of In_2_O_3_ nanocrystalline phase in the range 0–15 at. % Ga, while for higher Ga concentrations the films demonstrated an amorphous structure. The incorporation of Ga atoms into the host lattice during the growth in given technological conditions is an important finding regarding the high Ga solubility limit in ITO. TDTR measurements revealed a decreasing of room temperature thermal conductivity with increase of Ga-content from 2.0 Wm^−1^ K^−1^ at 5 at. % Ga down to 0.8 Wm^−1^ K^−1^ at 15 at. % Ga, while for higher Ga concentrations thermal conductivity saturates ~0.5–0.6 Wm^−1^ K^−1^. Theoretical modelling within DFT and BTE approaches demonstrated that this drop of thermal conductivity is primarily due to the redistribution of the metal atoms vibrations in the energy range 10–35 meV, accompanied by the appearance of multiple avoided-crossing modes throughout the Brillouin zone with decreased group velocity and shortened phonon mean free paths. In the range 15–20 at. % Ga both experimental and theoretical results confirm the evolution from propagative to diffusive phonon thermal transport in ITO:Ga nanofilms. The obtained results demonstrate the practical possibility of engineering the thermal conductivity in ITO films by Ga doping and may lead to their thermoelectric applications.

## Data Availability

All the data are reported in the paper directly.

## References

[1] Ohtaki M., Ogura D., Eguchi K., Arai H. (1994). High-Temperature thermoelectric properties of In_2_O_3_-based mixed oxides and their applicability to thermoelectric power generation. J. Mater. Chem..

[2] Zhu B., Zhang T., Luo Y., Wang Y., Tan T.T., Donelson R., Hng H.H., Li S. (2018). Improved densification and thermoelectric performance of In_5_SnSbO_12_ via Ga doping. J. Mater. Sci..

[3] Huang L., Guo J., Ge Z.-H., Jiang Y., Feng J. (2020). Significantly reduced lattice thermal conductivity and enhanced thermoelectric performance of In_2_O_3_(ZnO)_3_ ceramics by Ga_2_O_3_ doping. J. Solid State Chem..

[4] Bérardan D., Guilmeau E., Maignan A., Raveau B. (2008). In_2_O_3_:Ge, a promising n-type thermoelectric oxide composite. Solid State Commun..

[5] Lan J.-L., Liu Y., Lin Y.-H., Nan C.-W., Cai Q., Yang X. (2015). Enhanced thermoelectric performance of In_2_O_3_-based ceramics via nanostructuring and point defect engineering. Sci. Rep..

[6] Kim S., Kim D., Byeon J., Lim J., Song J., Park S., Park C., Song P. (2018). Transparent amorphous oxide semiconductor as excellent thermoelectric materials. Coatings.

[7] Liu Y., Xu W., Liu D.-B., Yu M., Lin Y.-H., Nan C.-W. (2015). Enhanced thermoelectric properties of Ga-doped In_2_O_3_ ceramics via synergistic band gap engineering and phonon suppression. Phys. Chem. Chem. Phys..

[8] Toberer E.S., Zevalkink A., Snyder G.J. (2011). Phonon engineering through crystal chemistry. J. Mater. Chem..

[9] Cocemasov A.I., Brinzari V.I., Nika D.L. (2020). Energetic, structural and electronic features of Sn-, Ga-, O-based defect complexes in cubic In_2_O_3_. J. Phys. Condens. Matter.

[10] Brinzari V., Nika D.L., Damaskin I., Cho B.K., Korotcenkov G. (2016). Thermoelectric properties of nano-granular indium-tin-oxide within modified electron filtering model with chemisorption-type potential barriers. Phys. E Low Dimens. Syst. Nanostruct..

[11] Bahk J.-H., Bian Z., Shakouri A. (2013). Electron energy filtering by a nonplanar potential to enhance the thermoelectric power factor in bulk materials. Phys. Rev. B.

[12] Brinzari V., Damaskin I., Trakhtenberg L., Cho B.K., Korotcenkov G. (2014). Thermoelectrical properties of spray pyrolyzed indium oxide thin films doped by tin. Thin Solid Films.

[13] Korotcenkov G., Brinzari V., Ham M.-H. (2018). In_2_O_3_-Based thermoelectric materials: The state of the art and the role of surface state in the improvement of the efficiency of thermoelectric conversion. Crystals.

[14] Frischbier M.V., Wardenga H.F., Weidner M., Bierwagen O., Jia J., Shigesato Y., Klein A. (2016). Influence of dopant species and concentration on grain boundary scattering in degenerately doped In_2_O_3_ thin films. Thin Solid Films.

[15] Brinzari V.I., Cocemasov A.I., Nika D.L., Korotcenkov G.S. (2017). Ultra-Low thermal conductivity of nanogranular indium tin oxide films deposited by spray pyrolysis. Appl. Phys. Lett..

[16] Balandin A., Wang K.L. (1998). Significant decrease of the lattice thermal conductivity due to phonon confinement in a free-standing semiconductor quantum well. Phys. Rev. B.

[17] Liu W., Asheghi M. (2006). Thermal conductivity measurements of ultra-thin single crystal silicon layers. J. Heat Transf..

[18] Boukai A.I., Bunimovich Y., Tahir-Kheli J., Yu J.-K., Goddard W.A., Heath J.R. (2010). Silicon nanowires as efficient thermoelectric materials. Materials for Sustainable Energy.

[19] Pawlak M., Jukam N., Kruck T., Dziczek D., Ludwig A., Wieck A.D. (2020). Measurement of thermal transport properties of selected superlattice and thin films using frequency-domain photothermal infrared radiometry. Measurement.

[20] Isacova C., Cocemasov A., Nika D.L., Fomin V.M. (2021). Phonons and thermal transport in Si/SiO_2_ multishell nanotubes: Atomistic study. Appl. Sci..

[21] Cahill D.G. (2004). Analysis of heat flow in layered structures for time-domain thermoreflectance. Rev. Sci. Instrum..

[22] Jiang P., Qian X., Yang R. (2018). Tutorial: Time-Domain thermoreflectance (TDTR) for thermal property characterization of bulk and thin film materials. J. Appl. Phys..

[23] Korotcenkov G., Brinzari V., Cerneavschi A., Cornet A., Morante J., Cabot A., Arbiol J. (2002). Crystallographic characterization of In_2_O_3_ films deposited by spray pyrolysis. Sens. Actuators B Chem..

[24] Brinzari V., Korotcenkov G., Golovanov V., Schwank J., Lantto V., Saukko S. (2002). Morphological rank of nano-scale tin dioxide films deposited by spray pyrolysis from SnCl_4_·5H_2_O water solution. Thin Solid Films.

[25] Korotcenkov G., Brinzari V., Boris I. (2008). (Cu, Fe, Co, or Ni)-Doped tin dioxide films deposited by spray pyrolysis: Doping influence on film morphology. J. Mater. Sci..

[26] Korotcenkov G., Cho B.K., Brinzari V. (2013). Spray pyrolysis of metal oxides SnO_2_ and In_2_O_3_ as an example of thin film technology: Advantages and limitations for application in conductometric gas sensors. Adv. Mater. Res..

[27] Alsaid D.A. (2012). Gravure Printability of Indium Tin Oxide Nanoparticles on Glass and PET Films for Applications in Printed Electronics. Ph.D. Thesis.

[28] Lide D.R. (2005). CRC Handbook of Chemistry and Physics.

[29] Chávez E., Cuffe J., Alzina F., Torres C.S. (2012). Calculation of the specific heat in ultra-thin free-standing silicon membranes. J. Phys. Conf. Ser..

[30] Jeong D.G., Ju H.I., Choi Y.G., Roh C.J., Woo S., Choi W.S., Lee J.S. (2019). Nanoscale heat transport through the hetero-interface of SrRuO_3_ thin films. Nanotechnology.

[31] Soler J.M., Artacho E., Gale J.D., García A., Junquera J., Ordejón P., Sánchez-Portal D. (2002). The SIESTA method for Ab Initio order-N materials simulation. J. Phys. Condens. Matter.

[32] Perdew J.P., Ruzsinszky A., Csonka G.I., Vydrov O.A., Scuseria G.E., Constantin L.A., Zhou X., Burke K. (2008). Restoring the density-gradient expansion for exchange in solids and surfaces. Phys. Rev. Lett..

[33] Hamann D.R., Schlüter M., Chiang C. (1979). Norm-Conserving pseudopotentials. Phys. Rev. Lett..

[34] Van Setten M.J., Giantomassi M., Bousquet E., Verstraete M.J., Hamann D.R., Gonze X., Rignanese G.-M. (2018). The PseudoDojo: Training and grading a 85 element optimized norm-conserving pseudopotential table. Comput. Phys. Commun..

[35] Monkhorst H.J., Pack J.D. (1976). Special points for brillouin-zone integrations. Phys. Rev. B.

[36] Marezio M. (1966). Refinement of the crystal structure of In_2_O_3_ at two wavelengths. Acta Crystallogr..

[37] Haynes W.M., Lide D.R., Bruno T.J. (2017). CRC Handbook of Chemistry and Physics.

[38] Togo A., Tanaka I. (2015). First principles phonon calculations in materials science. Scr. Mater..

[39] Mingo N. (2003). Calculation of Si nanowire thermal conductivity using complete phonon dispersion relations. Phys. Rev. B.

[40] Allen P.B., Feldman J.L. (1989). Thermal conductivity of glasses: Theory and application to amorphous Si. Phys. Rev. Lett..

[41] Li G., Yarali M., Cocemasov A., Baunack S., Nika D.L., Fomin V.M., Singh S., Gemming T., Zhu F., Mavrokefalos A. (2017). In-Plane thermal conductivity of radial and planar Si/SiO_x_ hybrid nanomembrane superlattices. ACS Nano.

[42] Agne M.T., Hanus R., Snyder G.J. (2018). Minimum thermal conductivity in the context of diffuson-mediated thermal transport. Energy Environ. Sci..

[43] Kishimoto K., Tsukamoto M., Koyanagi T. (2002). Temperature dependence of the seebeck coefficient and the potential barrier scattering of N-type PbTe films prepared on heated glass substrates by Rf sputtering. J. Appl. Phys..

[44] Preissler N., Bierwagen O., Ramu A.T., Speck J.S. (2013). Electrical transport, electrothermal transport, and effective electron mass in single-crystalline In_2_O_3_ films. Phys. Rev. B.

[45] Lee J.-H., Lee S.-H., Choi C., Jang S., Choi S. (2011). A review of thermal conductivity data, mechanisms and models for nanofluids. Int. J. Micro Nano Scale Transp..

[46] Renteria J.D., Ramirez S., Malekpour H., Alonso B., Centeno A., Zurutuza A., Cocemasov A.I., Nika D.L., Balandin A.A. (2015). Strongly anisotropic thermal conductivity of free-standing reduced graphene oxide films annealed at high temperature. Adv. Funct. Mater..

[47] Yoldas B.E., Partlow D.P. (1985). Formation of broad band antireflective coatings on fused silica for high power laser applications. Thin Solid Films.

[48] Du K., Deng S.P., Qi N., Zhou B., Chen Z.Q., Su X.L., Tang X.F. (2019). Ultralow thermal conductivity in In_2_O_3_ mediated by porous structures. Microporous Mesoporous Mater..

[49] Balandin A.A., Nika D.L. (2012). Phononics in low-dimensional materials. Mater. Today.

[50] El Sachat A., Alzina F., Sotomayor Torres C.M., Chavez-Angel E. (2021). Heat transport control and thermal characterization of low-dimensional materials: A review. Nanomaterials.

[51] Anufriev R., Nomura M. (2019). Coherent Thermal conduction in silicon nanowires with periodic wings. Nanomaterials.

[52] Cocemasov A.I., Isacova C.I., Nika D.L. (2018). Thermal transport in semiconductor nanostructures, graphene, and related two-dimensional materials. Chin. Phys. B.

[53] Christensen M., Abrahamsen A.B., Christensen N.B., Juranyi F., Andersen N.H., Lefmann K., Andreasson J., Bahl C.R., Iversen B.B. (2008). Avoided crossing of rattler modes in thermoelectric materials. Nat. Mater..

[54] Li W., Mingo N. (2015). Ultralow lattice thermal conductivity of the fully filled skutterudite YbFe_4_Sb_12_ due to the flat avoided-crossing filler modes. Phys. Rev. B.

[55] Baggioli M., Cui B., Zaccone A. (2019). Theory of the phonon spectrum in host-guest crystalline solids with avoided crossing. Phys. Rev. B.

